# Dynamics of among-individual behavioral variation over adult lifespan in a wild insect

**DOI:** 10.1093/beheco/arv048

**Published:** 2015-04-29

**Authors:** David N. Fisher, Morgan David, Tom Tregenza, Rolando Rodríguez-Muñoz

**Affiliations:** ^a^Centre for Ecology and Conservation, University of Exeter, Treliever Road, Penryn TR109FE, UK and; ^b^Department of Biology-Ethology, University of Antwerp, Drie Eiken Campus, Universiteitsplein 1, 2610 Wilrijk (Antwerpen), Belgium

**Keywords:** animal personality, behavioral reaction norms, *Gryllus*, plasticity, wild crickets.

## Abstract

Investigating patterns of among and within-individual trait variation in populations is essential to understanding how selection shapes phenotypes. Behavior is often the most flexible aspect of the phenotype, and to understand how it is affected by selection, we need to examine how consistent individuals are. However, it is not well understood whether among-individual differences tend to remain consistent over lifetimes, or whether the behavior of individuals relative to one another varies over time. We examined the dynamics of 4 behavioral traits (tendency to leave a refuge, shyness, activity, and exploration) in a wild population of field crickets (*Gryllus campestris*). We tagged individuals and then temporarily removed them from their natural environment and tested them under laboratory conditions. All 4 traits showed among-individual variance in mean levels of expression across the adult lifespan, but no significant differences in how rapidly expression changed with age. For all traits, among-individual variance increased as individuals got older. Our findings reveal seldom examined changes in variance components over the adult lifetime of wild individuals. Such changes will have important implications for the relationship between behavioral traits, life-histories, and fitness and the consequences of selection on wild individuals.

## Lay Summary

Wild crickets show consistent patterns of behavior over their adult lifetimes and as they get older they become increasingly predictable. We tagged crickets and then periodically recaptured them and measured their behavior in the lab. This revealed that rather than variation in how age affects behavior, there were consistent patterns across the whole population. We do not have a situation where some crickets live fast and die young while others take it easy and slow.

## INTRODUCTION

Explaining variation in wild populations is crucial to the study of evolution. Evolutionary and ecological studies have often considered behavioral variation among individuals in a single population to be noise surrounding adaptive peaks (Wilson 1998). However, recent studies have emphasized that such among-individual behavioral variation is persistent and likely to be adaptive (Wilson 1998; Dall et al. 2004; Smith and Blumstein 2008; Bell et al. 2009). Consistent among-individual behavioral variation has classically been studied in traits such as boldness-shyness, exploration, aggressiveness, or activity. These traits, often referred to as “personality traits,” are thought to reflect underlying tendencies and hence are expected to influence other behaviors across contexts in a consistent way (Réale et al. 2007). However, an individual’s phenotype has the potential to exhibit plasticity over its lifetime (Nussey et al. 2007). Therefore, adaptive explanations for the maintenance of consistent behavioral variation among individuals must deal with potential age-related behavioral variation within individuals (Dingemanse and Réale 2005; Schuett et al. 2010; Kight et al. 2013).

Two key aspects of behavioral consistency are among-individual differences (i.e., the extent to which an individual is reliably different from other individuals) and the absolute variation (or lack of variation) in a trait over time within individuals (also known as repeatability and stability; David et al. 2012). Few studies have examined how these variance components change over time. Human behavior is known to become more consistent with age, with reinforcement of behavior suggested as a key mechanism (Roberts and Del Vecchio 2000), but studies on nonmorphological or life-history traits in wild animals are less common (see Wilson et al. 2008 and Brommer 2013a for reviews). Work on *Drosophila* has indicated that additive genetic variance in fecundity follows a U-shaped curve, with lowest variance at intermediate ages (Tatar et al. 1996), while mortality shows the opposite pattern (Promislow et al. 1996). Furthermore, heritability in laying date of wild mute swans (*Cygnus olor*) also followed a U-shaped pattern (Charmantier et al. 2006), with highest heritability at the oldest ages. Dingemanse et al. (2012) measured exploratory behavior over time in great tits (*Parus major*) and found that, in 4 separate populations, among-individual variance was higher in repeated tests compared to an initial test. They did not however model whether variance changes continually with individual age, which would allow us to assess whether a continual increase or the U-shaped pattern exists as found in the above studies.

A meta-analysis by Bell et al. (2009) on the repeatability of behavior found that juveniles are more consistent than adults. However, there was no comparison for different stages of the adult life, yet, as this is when reproduction takes place it is when the heritability of traits is relevant. Repeatability is related to heritability (but does not necessarily set the upper limit; Dohm 2002) and so influences the expected response to selection. If repeatability estimates do differ depending on the age of the organism, then measuring at one point in time may not adequately reflect the true repeatability of that trait. This could lead to overestimates for response to selection and rate of evolutionary change (Charmantier et al. 2006). What is required are repeated estimates of variance components over the adult lifetime of individuals. Furthermore, recent reviews have highlighted how individuals within a population may not all respond to environmental change or aging in the same way (Nussey et al. 2007; Dingemanse et al. 2010). If individuals change with age differently, their level of behavior relative to others can differ depending on the age they are measured at (Brommer 2013b; Kluen and Brommer 2013). The study of relationships between the trait and other behavioral or life-history traits that are more stable over time is common (e.g., Dingemanse et al. 2004; Brown et al. 2005; Minderman et al. 2009; Amy et al. 2010; Cole and Quinn 2012; Patrick et al. 2012; Adriaenssens and Johnsson 2013; Aplin et al. 2013; Bouwhuis et al. 2014). However, if correlations or rank orders of behaviors change over time, then detecting such associations will entirely depend on when measures are taken and could give wildly different results (Kluen and Brommer 2013). For instance, Wolf et al. (2007) predict that individuals with high future reproductive fitness will take fewer risks, while Luttbeg and Sih (2010) predict that risk taking and reproductive success are in fact positively related. Teasing these hypotheses apart without traits that have rank-order stability over the reproductive lifetime of individuals is difficult, yet crucial if we are to understand the evolution of animal personalities.

Measurement over the adult lifespan requires the individual to be identified when it becomes adult, and followed continually afterwards. This is hard to do in wild animals (but see: Martin and Réale 2008; Réale et al. 2009; Dingemanse et al. 2012; Aplin et al. 2013), hence studies looking at behavioral change over lifetimes have tended to use animals raised in captivity (e.g., Sinn et al. 2008; Herde and Eccard 2013). However, accidental selection by breeding populations of study organisms in captivity could lead to unnatural decreases or increases in among-individual variation or mean levels of behavior quite different from that expressed in the wild (McDougall et al. 2006; Archard and Braithwaite 2010). Even those animals that are offspring of wild parents may show unusual developmental trajectories due to captivity conditions (Archard and Braithwaite 2010). Therefore, studies on wild populations are crucial. Additionally, examining adaptive behaviors in captivity has been criticized, as individuals in novel settings may behave in novel ways that have not been subject to selection (Niemelä and Dingemanse 2014). However, previous research has shown relationships between personality traits measured in captivity and competitive ability (Cole and Quinn 2012), fitness (Dingemanse et al. 2004; Adriaenssens and Johnsson 2013) and rate of promiscuity (Patrick et al. 2012) all in the wild. Furthermore, Herborn et al. (2010) demonstrate that personality traits in captivity predict analogous traits in the wild. Therefore, capturing wild animals and testing them in captivity before returning them to the wild seems an appropriate compromise between validity and practicality.

The paucity of repeated measures on wild animals’ behavior is especially true for invertebrates, which are often difficult to study in the wild because of their small size (but see Briffa and Greenaway 2011; Pinter-Wollman et al. 2012). However, insects such as field crickets are good candidates for the study of behavior in wild animals, due to their regular use of particular burrows which serve as refuges from predation (Rodríguez-Muñoz et al. 2011). This makes it possible to tag individuals and locate them in the environment by monitoring their burrows (Rost and Honegger 1987; Hissmann 1990; Rodríguez-Muñoz et al. 2010; Rodríguez-Muñoz et al. 2011). Moreover, various field cricket species have been extensively used in behavioral studies conducted in the laboratory (Hedrick 2000; Hedrick and Kortet 2006, 2011; Niemelä et al. 2012a, 2012b; Tyler et al. 2013).

Here, we study the behavior of a population of the European field cricket *Gryllus campestris* in the wild. We captured all the members of an isolated population, tagged them, and released them back into the field. At the same time, we assayed their behavior for 4 traits. Subsequently, we regularly recaptured and re-tested them, to examine how the repeatability of those traits changed over time and if behavior of individuals in relation to each other varies over their adult lifetime. We also investigated correlations among behaviors at both the among- and within-individual level. Separating phenotypic correlations into these 2 components is essential to avoid erroneous conclusions about correlations, as among-individual correlations can mask within-individual correlations (David et al. 2014). Furthermore, it allows us to determine whether there is potential for correlated plasticity of traits (Dingemanse and Dochtermann 2013; Brommer 2013c), which suggests that traits form “evolutionary characters” (Araya-Ajoy and Dingemanse 2014). We predict that patterns of among-individual variance will follow similar patterns to that previously observed in behavioral traits and so increase with age (Roberts and Del Vecchio 2000; Dingemanse et al. 2012) and this to be reflected in estimates of repeatability. We then predict that individuals will be stable over their lifetime in the level traits relative to each other (see Fig. 3 in Brommer 2013a). Finally, a meta-analysis suggests that the direction of correlations is typically weakly positive (Garamszegi et al. 2012) so we refrain from making any strong predictions regarding the correlations, especially as few studies have split phenotypic correlations into both among and within-individual correlations.

## METHODS

### Study subjects

The study was carried out at the “WildCrickets” project field site in Northern Spain (Rodríguez-Muñoz et al. 2010; Bretman et al. 2011). We collected data during April-June when adults of this univoltine species are active, in 2013 and 2014. Data from each year are pooled and differences modeled using year as a fixed effect (see Statistical analysis below). Using a network of video cameras (120 in 2013 and 133 in 2014) and regular direct observations for non-video monitored burrows, we were able to determine the occupation of burrows by nymphs, and the adults’ emergence dates. We began trapping and testing newly emerged adults 3 days after their emergence date (3.33±3.76, mean ± SD). Subsequently, we re-caught and re-tested each individual cricket at time intervals of around 10 days, starting from the date of the first postemergence test and continuing until the individual was no longer observed alive.

Trapping typically ran from 08:00 to 16:00 GMT. Once caught, we placed the crickets in individual 150-mL plastic containers then transferred them to the cricket processing area in a building 30 m away from the centre of the meadow. Traps were checked every 15min to ensure crickets did not languish in the trap. Once a cricket was caught, to prevent other animals (including other crickets) from taking over the burrow, the trap was left blocking the entrance while the cricket was being tested. We tested crickets on the day they were caught, placing them in controlled temperature room at 20.12±0.82 °C (mean ± SD) for video observation. The total time a cricket was in captivity was 90–120min, with an additional 40min at first capture for the tagging procedure.

### Experimental setup

We conducted tests in 16 open-topped plastic boxes 290×201×212mm, with a piece of A4 paper lining the bottom. The paper was replaced between consecutive tests and the boxes wiped first with soapy water and then ethanol to remove any pheromones released by previous crickets. We monitored each box from above with a fixed camera, connected to a computer in the adjacent cricket processing area. We used software designed for CCTV surveillance, which has been extensively customized by the developer to facilitate its use in biological research (iCatcher ver. 5.2, www.icode.co.uk/icatcher). We tracked and recorded the movements of each cricket during the tests. This allowed up to 16 crickets to be tested simultaneously. Before the start of the test, we placed the focal crickets in opaque cylindrical tubes (80×20mm), with detachable lids at either end. We placed each sealed tube on its side into one of the boxes against the centre of one end of the box, with the head of the cricket facing towards the centre of the box. Once we had placed all crickets in position, we removed the lids at the same end as the crickets’ heads and left the room. We recorded the exact time of lid removal for each focal cricket, but within a test these differed by less than 1min. Tests ran for 30min.

After a 30-min test, we returned crickets to their 150-mL plastic containers and left them in an isolated room for another 30min. We then repeated the test described above, placing crickets into boxes irrespective of the box they had previously been tested in. After these 2 tests, we weighed and tagged newly emerged adults (see Rodríguez-Muñoz et al. 2010 for details). We released tagged adults by returning them to the burrow from which they had been collected, ensuring that they re-entered the burrow. Total handling time was similar (within 30min) for all crickets being tested as we could test one set of individuals during the 30-min isolation period of another set. Unless otherwise stated, for all analyses, we only used the first of the 2 tests carried out for each day the cricket was captured. We also ran analyses for the less conservative approach of using the second trial to measure traits that required a cricket to leave the tube when the cricket failed to emerge on the first trial and found qualitatively similar results.

Adult male crickets start singing (a shrill sound made by rubbing the fore-wings together to attract mates) a few days postemergence, indicating that they are sexually active. This could alter the perceived environment for crickets in neighboring boxes. To prevent bias resulting from occasional singing by one or more of the adult males being tested, we played a recording of 4 male crickets singing throughout tests that involved sexually active adult crickets. This recording was made in the same meadow at a similar temperature to the experimental conditions. This procedure standardized the auditory environment to one in which song from ≥4 males was always present.

### Data collection

We examined 4 behaviors. First, tendency to leave the tube, with whether or not the cricket left the tube at all in the 30min as a binary response variable. Second, position on the boldness-shyness continuum was measured as “shyness” and defined as the latency between the start of the test and the time when the cricket’s head emerged from the tube (Hedrick 2000; Hedrick and Kortet 2006, 2011; Niemelä et al. 2012a, 2012b). This measure is strongly correlated with the time until the entire body leaves the tube (Hedrick and Kortet 2006) and the time until a cricket becomes active inside the tube (Niemelä et al. 2012b). The third behavior was activity: general tendency to move around (Réale et al. 2007; Tremmel and Muller 2012). Using iCatcher, we set 8 unique virtual trip wires across the box in a lattice, 4 vertical and 4 horizontal, with each wire covering half the length or width of the box, giving the appearance of a 3 by 3 square grid. Once the cricket’s head emerged from the tube, iCatcher counted the number of times the cricket crossed any wire after it had emerged from the tube. An individual’s activity score was the number of trip wires it crossed divided by the time it spent outside of the tube before the end of the test, that is the rate of wires crossed. This was multiplied by 100 and rounded to give integers for use in models with a Poisson distribution error structure. Finally, we quantified exploratory behavior (Réale et al. 2010). This was the number of unique trip wires a cricket crossed in the first minute after it had left the tube. This represents the crickets’ tendency to visit different areas of the box rather than repeatedly being active in one corner.

These 4 traits were chosen as having the potential to be to ecologically relevant to our study population. Both sexes build and spend a lot of time at burrows, which are used as a refuge from predators, while also moving among other burrows in the field to find potential mates. Therefore, our measures of tendency to leave the tube and shyness closely mimic the behavior crickets express in the field when leaving their burrows, while activity and exploration reflect general movement among burrows and willingness to visit new burrows, respectively.

### Statistical analyses

We conducted all analyses in R ver. 3.0.2 (R Core Team 2014), using the package Markov Chain Monte Carlo methods for Generalised Linear Mixed Models (MCMCglmm) (Hadfield 2010b). First, we constructed random regression (RR) models (also known as random slope models) for each trait (Nussey et al. 2007; Dingemanse et al. 2010). RRs model each trait as being linearly predicted by age for each individual, as in Edenbrow and Croft (2011). We only consider a linear relationship, as the estimation of high-order polynomials requires a larger number of measures per individual than we could collect. Each individual has an intercept and slope of a regression line, so we can estimate the among-individual variation in these intercepts and slopes as well as estimate the intercept–slope correlation (Nussey et al. 2007; Dingemanse et al. 2010). RRs fit individual functions of continuous covariates (in our case, age) as random effects (Henderson 1982) and have been extensively used to investigate ontogeny (e.g., estimating individual growth curves; Wilson et al. 2005). Following Martin et al. (2011), we retained individuals that only recorded traits scores on one trial, as although they cannot contribute to estimates of variance among slopes they can contribute to estimates of variance among intercepts. When the covariate has been centered, significant among-individual variation in the intercepts of the RRs indicates that individuals consistently differ in the trait at the mean value of the covariate. Significant among-individual variation in slopes indicates that individuals change with age differently. A significant intercept–slope interaction indicates that an individual’s mean trait level is related to the way the trait changes with age. Furthermore, RRs can be used to estimate the change in among-individual variance over time (Brommer 2013b). This gives an estimate of the continuous change in among-individual variance over time, which we plot for each trait.

We also wished to get point estimates for both among-individual and residual variance across the adult lifespan of our individuals. As described above, each time we captured the crickets they were tested twice, and for this analysis this second test is also used. Therefore, we can calculate point estimates for the repeatability of cricket behavior for the first capture and each subsequent recapture. To avoid confounding effects from the possibility that individuals that die at a young age are overrepresented at early ages and may also differ systematically from longer lived individuals, we only included crickets that at some point were re-caught at 30 days old or older. To estimate the among-individual and residual variance for each capture, an interaction between ID and capture number (1–4) was included in the among-individual and residual covariance structure of the mixed model, giving separate estimates for among-individual and residual variance for each of the captures. There were in fact 4 crickets who were capture a fifth time. However, this is not a great enough number to estimate the variance components with any confidence, so these tests (but not the other test for these crickets) were excluded. We calculated repeatability for non-Gaussian data following Nakagawa and Schielzeth (2010), using their definition of repeatability as the proportion of variance that is reproducible across repeated measures of an individual. We calculate repeatability scores from the posterior distributions of among-individual and residual variances and provide the mode (posterior distribution mode [PDM]) and the 95% credible intervals (CRIs) of the resulting distributions.

To determine whether shyness, activity, and exploration were correlated with one another, we built a multivariate mixed-effect model. We could not include tendency to leave the tube in this model as all 3 traits required a cricket to leave the tube. We extracted the among-individual and residual variances and covariances for the traits, allowing us to calculate the among- and within-individual correlations (Hadfield 2010b; Dingemanse and Dochtermann 2013; Brommer 2013c). Correlations are calculated by dividing the covariance of the traits in question by the square root of the product of their variances and judged important if the 95% CRIs do not cross 0. The fixed effects in each model were sex (males as the contrast), age (number of days from emergence date), laboratory temperature (°C), mass at first capture (grams), and year (a 2-level factor, 2013 or 2014, with 2014 as the contrast). Age, mass, and temperature were all mean centered. We also included the test number as a fixed effect, to allow us to estimate and control for any habituation effects. Finally, we also included the maximum age at which that cricket was ever tested as a fixed effect, also mean centered. This allowed us to model the effect of selective disappearance, and so measure both within-individual change with age and among-individual difference between ages (van de Pol and Verhulst 2006). In the multivariate model, the fixed effects were modeled to have separate effects on each response variable. The modes of the PDMs and the CRIs for random and fixed effects from each model are given in the Tables 1–4. The effect of a factor is modeled as a frequency distribution of effect strengths. The importance of among-individual variance in intercepts and slopes and residual variance is judged by the distance from 0 and the spread of the 95% CRIs. If the variance is truly 0, the CRIs will have 0 coverage (Higgins and Thompson 2002), so narrow CRIs near 0 indicate a lack of variance in that component. Importance of the fixed effects and covariances is judged by whether the CRIs overlap 0. Alongside the CRIs, the fixed effects in the models can be evaluated using pMCMC values; pMCMC values that are near 1 indicate a lack of influence of the fixed effect. Tendency to leave the tube was modeled with an ordinal error structure, a logit link function and additive errors. The residual variance cannot be estimated simultaneously with the among-individual variance in a model with a binary response as it is wholly described by the mean, so we fix it to one (Hadfield 2010a). Shyness and activity were modeled with a Poisson distribution, a log-link function and additive errors and exploration with a Gaussian distribution and additive errors. As the tendency to cross 0 trip wires is modeled in the analyses for activity, those scores are removed for the analyses of exploration. This allows us to fit a Gaussian distribution; without removing the zeros, the distribution is not amenable to analysis. Models were assessed for appropriate mixing and smooth posterior distributions of effects. Priors were made less informative and number of iterations increased until satisfactory model plots were obtained.

**Table 1:** The tendency of the crickets to leave the tube

Component	PDM	Lower 95% CRI	Upper 95% CRI	pMCMC
*V*_A_ in intercepts	0.680	0.304	1.353	NA
*V*_A_ in slopes	0.002	<0.001	0.006	NA
Intercept–slope covariance	0.009	−0.022	0.032	NA
Intercept–slope correlation	0.058	−0.344	0.922	NA
Model intercept	1.066	0.484	1.798	NA
***Age***	**0.060**	**0.024**	**0.093**	**0.001**
*Mass*	−0.007	−0.933	1.124	0.863
***Temperature***	**0.433**	**0.296**	**0.651**	**<0.001**
*Sex*	−0.219	−0.566	0.015	0.077
*Test*	−0.095	−0.313	0.106	0.386
*Maximum age*	−0.007	−0.019	0.003	0.176
*Year*	−0.180	−0.471	0.158	0.318

Given are the PDMs and the 95% CRIs. *V*_A_ refers to among-individual variance. In models with a binary response, when estimating the among-individual variance, the residual variance is entirely defined by the mean (Hadfield 2010a), and so it is not given. Fixed effect names are italicized, those that have 95% CRIs that do not cross 0 are also in bold. pMCMC scores are not applicable (NA) to variance components as they are constrained to be above 0. NA is also given for the model intercept as the null hypothesis for this test is that the intercept is 0, which is not biologically relevant.

**Table 2:** Results of RR models for shyness, activity, and exploration

Trait	Component	PDM	Lower 95% CRI	Upper 95% CRI	pMCMC
Shyness	*V*_A_ in intercepts	0.125	0.030	0.238	NA
	*V*_A_ in slopes	0.003	0.003	0.004	NA
	Intercept–slope covariance	−0.001	−0.006	0.004	NA
	Intercept–slope correlation	−0.044	−0.280	0.144	NA
	Model intercept	5.570	5.227	5.936	**NA**
	***Age***	**−0.018**	**−0.041**	**−0.003**	**0.021**
	*Mass*	−0.227	−0.862	0.411	0.479
	***Temperature***	**−0.162**	**−0.263**	**−0.052**	**0.004**
	*Sex*	−0.187	−0.341	0.0127	0.074
	*Test*	0.054	−0.083	0.159	0.558
	*Maximum age*	0.003	−0.005	0.009	0.651
	***Year***	**−0.360**	**−0.565**	**−0.090**	**0.01**
	Residual variance	1.010	0.843	1.152	NA
Activity	*V*_A_ in intercepts	1.778	1.544	2.083	NA
	*V*_A_ in slopes	0.001	−0.013	−0.003	NA
	Intercept–slope covariance	−0.008	−0.013	−0.003	NA
	Intercept–slope correlation	−0.491	−0.696	−0.250	NA
	Model intercept	0.056	−0.015	0.097	NA
	***Age***	**0.032**	**0.018**	**0.047**	**<0.001**
	*Mass*	0.173	−0.421	0.790	0.557
	***Temperature***	**0.286**	**0.189**	**0.368**	**<0.001**
	***Sex***	**−0.172**	**−0.325**	**−0.015**	**0.026**
	*Test*	−0.011	−0.113	0.064	0.712
	*Maximum age*	−0.006	−0.013	<0.001	0.097
	*Year*	−0.247	−0.482	0.073	0.093
	Residual variance	0.480	0.367	0.615	NA
Exploration	*V*_A_ in intercepts	0.517	0.047	1.165	NA
	*V*_A_ in slopes	0.006	0.004	0.008	NA
	Intercept–slope covariance	−0.012	−0.038	0.004	NA
	Intercept–slope correlation	−0.228	−0.514	0.058	NA
	Model intercept	3.927	3.171	4.593	NA
	*Age*	0.019	−0.018	0.052	0.375
	*Mass*	0.565	−0.898	1.773	0.533
	***Temperature***	**0.483**	**0.227**	**0.671**	**<0.001**
	*Sex*	−0.062	−0.433	0.344	0.814
	*Test*	−0.090	−0.321	0.130	0.484
	*Maximum age*	0.004	−0.013	0.019	0.734
	***Year***	**0.684**	**0.172**	**1.104**	**0.007**
	Residual variance	3.570	2.858	4.222	NA

The relative importance of among-individual variance (*V*_A_) in intercepts and slopes, and residual individual variance is judged by the distance of the PDM from 0 and the spread of the 95% CRIs. Fixed effects are italicized, those that have 95% CRIs that do not cross 0 are also in bold. pMCMC scores are not applicable (NA) to variance components as they are constrained to be above 0. NA is also given for the model intercept as the null hypothesis for this test is that the intercept is 0, which is not biologically relevant.

**Table 3:** Results for separate univariate mixed-models of shyness, activity, and exploration, with different variances estimated for the first capture (test 1) and 3 subsequent recaptures (tests 2–4)

Trait	Component	Capture number	PDM	Lower 95% CRI	Upper 95% CRI
Tendency to leave the tube	Among- individual variance	1	2.871	1.090	5.972
		2	1.531	0.494	3.439
		3	1.973	0.655	4.686
		4	1.124	<0.001	4.676
	Repeatability	1	0.500	0.304	0.683
		2	0.380	0.159	0.540
		3	0.495	0.236	0.630
		4	0.361	0.033	0.632
Shyness	Among-individual variance	1	0.313	<0.001	0.815
		2	0.285	<0.001	0.500
		3	0.349	0.140	0.702
		4	0.002	<0.001	0.701
	Residual variance	1	0.955	0.682	1.557
		2	1.000	0.748	1.304
		3	0.950	0.759	1.293
		4	1.54	1.106	2.164
	Repeatability	1	0.351	<0.001	0.492
		2	0.165	<0.001	0.358
		3	0.289	0.113	0.466
		4	0.002	<0.001	0.341
Activity	Among-individual variance	1	0.404	<0.001	1.011
		2	0.186	0.080	0.346
		3	0.197	0.102	0.361
		4	0.187	0.093	0.312
	Residual variance	1	0.399	0.162	0.912
		2	0.226	0.134	0.370
		3	0.208	0.130	0.355
		4	0.054	0.030	0.111
	Repeatability	1	0.504	0.060	0.721
		2	0.330	0.160	0.504
		3	0.339	0.192	0.513
		4	0.460	0.276	0.597
Exploration	Among-individual variance	1	0.007	<0.001	1.425
		2	1.246	<0.001	2.165
		3	0.007	<0.001	1.663
		4	0.010	<0.001	1.777
	Residual variance	1	3.036	2.013	4.349
		2	3.134	2.388	4.59
		3	3.153	2.451	4.574
		4	3.436	2.53	5.027
	Repeatability	1	0.002	<0.001	0.379
		2	0.292	<0.001	0.436
		3	0.002	<0.001	0.370
		4	0.002	<0.001	0.379

Given are the PDM and the 95% CRIs. Fixed effects were included in the model but not shown here as the confidence will be inflated by the use of both tests per capture.

**Table 4:** Results for multivariate mixed-model of shyness, activity, and exploration

Component	Trait(s)	PDM	Lower 95% CRI	Upper 95% CRI
Among-individual variances	Shyness	0.199	0.060	0.345
	Activity	0.080	0.033	0.125
	Exploration	0.130	0.034	0.722
Among-individual covariances	Shyness and activity	−0.024	−.076	
	0.044			
	Shyness and exploration	0.050	−0.143	0.206
	Activity and exploration	−0.022	−0.109	0.102
Among-individual correlations	Shyness and activity	−0.153	−0.669	0.327
	Shyness and exploration	0.179	−0.466	0.834
	Activity and exploration	0.064	−0.772	0.575
Residual variances	Shyness	1.182	0.990	1.364
	Activity	0.131	0.080	0.171
	Exploration	4.125	3.586	4.780
Residual covariances	Shyness and activity	−0.031	−0.090	0.045
	Shyness and exploration	−0.586	−0.839	−0.360
	Activity and exploration	0.334	0.217	0.469
Residual correlations	Shyness and activity	−0.054	−0.238	0.113
	**Shyness and exploration**	**−0.288**	**−0.370**	**−0.167**
	**Activity and exploration**	**0.501**	**0.337**	**0.629**

Given are the PDM and 95% CRIs of the covariances or correlations estimated. Correlations are considered significant if the 95% CRIs do not cross 0, highlighted in bold. Due to length, the fixed effects are not presented here but are available in the Supplementary Material.

## RESULTS

### Among- and within-individual change with age and change in among-individual variance with age

In total, we performed 2474 assays over 582 individuals. Of these, 1248 came from the first test of a recapture and were used in the RR to model the tendency for a cricket to leave the tube. This is slightly more than half the total number of tests as some crickets were not tested twice at a recapture due to time constraints. Results from this model are presented in Table 1. In summary, there was substantial among-individual variance in intercepts, but not in slopes, and the intercept–slope correlation was not significant. Crickets were more likely to leave the tube as they aged and when it was warmer. The 95% CRIs for the rest of the fixed effects overlapped 0, although males showed a tendency to be less likely to leave the tube. There was no evidence for selective disappearance, so crickets with longer adult lifespans did not have a different tendency to leave the tube to crickets with short adult lifespans.

The among-individual change with age (= within-individual change with age [fixed effect of age] + effect of selective disappearance [fixed effect of maximum age]; van de Pol and Verhulst 2006) was of the same sign as the within-individual change with age. This indicates that an individual was more likely to leave the tube as it aged and older adults are more likely to leave the tube than younger adults. This correspondence in sign between the among-individual change and the within-individual change was found for all the traits we examined. Mass did not influence tendency to leave the tube. Crickets showed no evidence of habituation as the effect of test substantially overlapped 0, and crickets were equally likely to leave the tube in each year.

In 61% of the above assays, crickets left the tube. These measures were used for the RRs for shyness and activity. Of these 763 assays, a cricket crossed 0 trip wires in 1min 22% of the time, leaving 596 measures for the RR of exploration. The results from these RRs are presented in Table 2.

**Figure 1:**
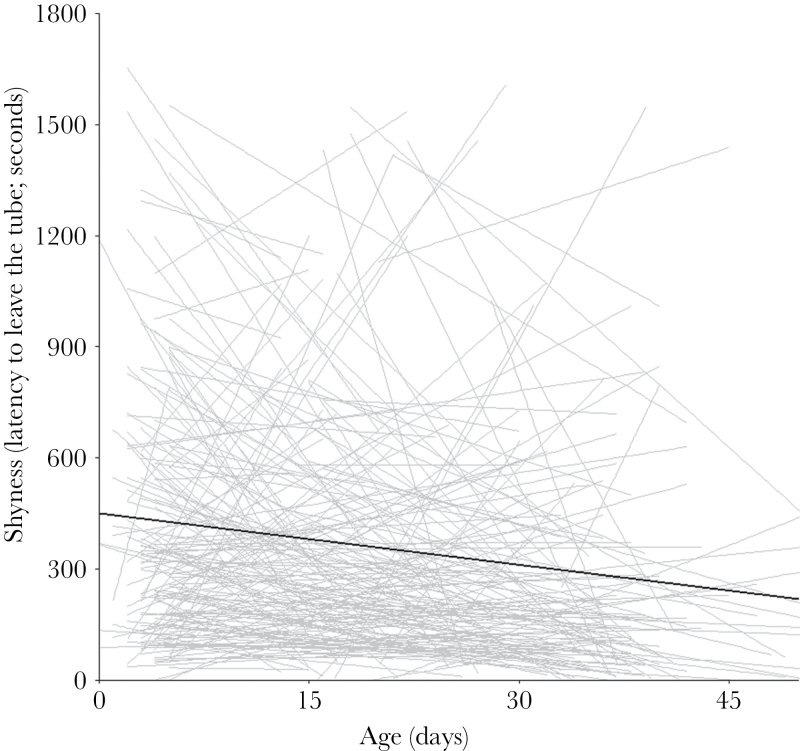
Individual plots of shyness change with age, with a linear trend line through each individual’s data points in grey and a population line in black. Shyness showed an important degree of among-individual variation in intercepts, but not in slopes and there was no intercept–slope correlation (*r* = −0.044). The fixed effect of age was negative, so individuals decreased their shyness with age (see Table 2 for full results).

For shyness (Figure 1), there was among-individual variance in intercepts, so individuals showed consistent differences in shyness, but there was again little among-individual variance in slopes and no intercept–slope correlation. Cricket left the tube faster when older, at higher temperatures, and in 2014. Males showed a nonsignificant tendency to be shyer than females. Again there was no evidence of habituation, selective disappearance, and no effect of mass.

**Figure 2:**
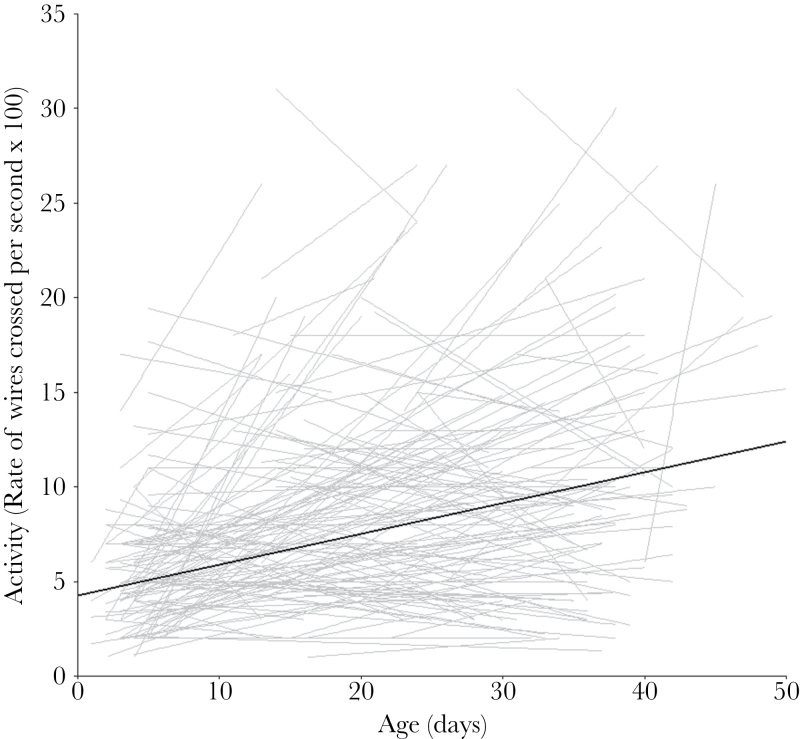
Individual plots of activity change with age, with a linear trend line through each individual’s data points in grey and a population line in black. Activity showed an important degree of among-individual variation in intercepts, but not in slopes. There was a negative intercept–slope correlation (*r* = −0.491). Individuals increased their activity level with age (see Table 2 for full results).

There was among-individual variance in intercepts for activity (Figure 2), but little in slopes, and there was a significant, negative intercept–slope correlation. Crickets were more active when older and at hotter temperatures, and males were less active than females. Otherwise there was no effect of selective disappearance, mass, year, or habituation.

**Figure 3:**
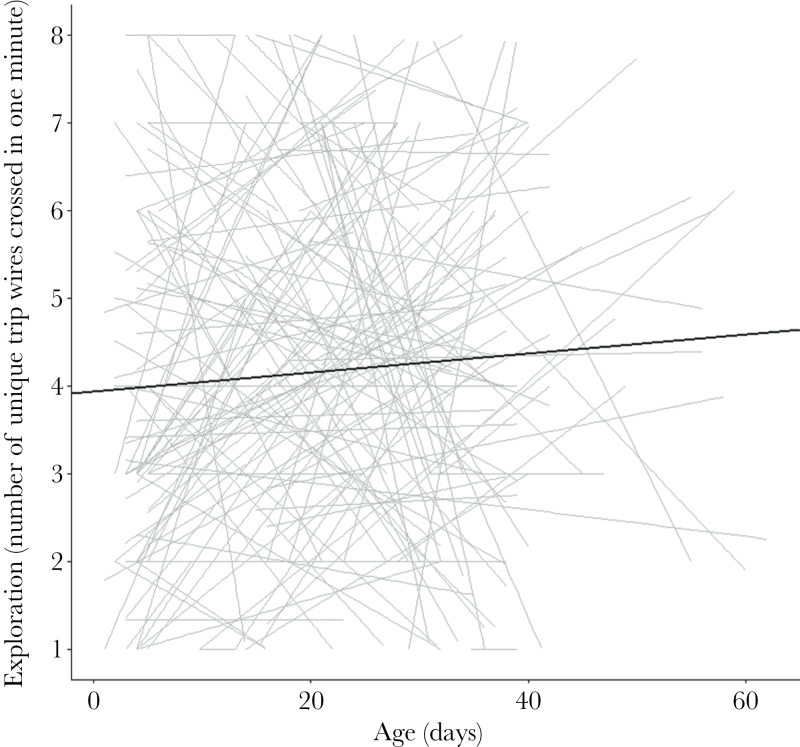
Individual plots of exploration change with age, with a linear trend line through each individual’s data points in grey and a population line in black. Exploration showed among-individual variation in intercepts, but not in slopes and there was no intercept–slope correlation (*r* = −0.228). The fixed effect of age was nonsignificant, so the population did not tend to change in exploration with age (see Table 2 for full results).

For exploration (Figure 3), there was among-individual variance in intercepts, little in slopes, and no intercept–slope correlation. Exploration increased with temperature and was higher in 2014 than in 2013. Males showed a nonsignificant tendency to be less exploratory. Crickets were more exploratory in 2014. Otherwise there was no effect of selective disappearance, mass, or habituation.

**Figure 4:**
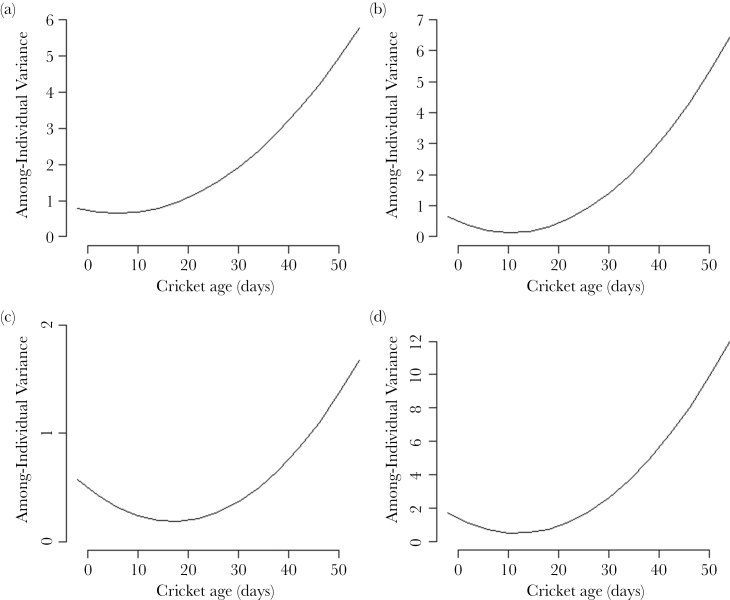
Plots of the among-individual change with age. (a) Tendency to leave the tube, (b) shyness, (c) activity, (d) exploration. Values calculated from the results of the RRs following code provided by (Brommer 2013b).

All traits showed a similar pattern in the change of the among-individual variance with age. There was a small decrease in among-individual variance from the youngest to the mean age, then a large increase through to old age. This appears to be a mixture of a steady increase and a U-shaped curve (Figure 4a–d).

### Differences in variance estimates across recaptures

Across tests 1–4, there were 318, 339, 310, and 150 measures from crickets that survived to be tested at greater than 30 days old, including both tests from each capture. Average ages at each recapture were 4.2, 21, 32, and 40 for tests 1–4. For crickets that left the tube, and so were included in the analyses for shyness and exploration, there were 135, 247, 242, and 133 measures for tests 1–4. The average ages over tests 1–4 for this subset was 4.9, 21, 32, and 41. There were 99, 210, 200, and 123 measures of a non-zero exploration score across tests 1–4. Average ages in this subset was 5.2, 21, 32, and 41. Estimates of among-individual and residual variance, along with repeatability estimates for each of the 4 traits, are presented in Table 3. Tendency to leave the tube showed repeatability between 0.36 and 0.5, with no clear pattern across captures. Shyness only had a repeatability above 0 (based on the lower 95% CRIs) once, for crickets in the third capture (0.29). Activity showed a U-shaped pattern in repeatability, with highest scores at the first (0.50) and last (0.46) test. These were not however significantly different from the estimates at the intermediate tests (0.33 and 0.34 for second and third tests, respectively). At no capture did crickets show non-zero repeatability for exploration. Fixed effects were included in the models but are not shown as the confidence of the estimate will be inflated by including both tests for a given capture.

### Behavioral correlations

To incorporate exploration, we used the 596 measures of shyness and activity that corresponded to the non-zero exploration scores used in the RR for exploration. None of the among-individual correlations were significant (Table 4). Exploration showed a significant negative residual correlation with activity, and a significant negative correlation with shyness. Activity and shyness showed no residual correlation. Fixed effects for this model are shown in the supplementary materials due to length (Supplementary Table S1).

## DISCUSSION

### Among-individual variance in intercepts, but not in slopes

Across all traits, we found substantial among-individual variance in behavior at mean ages among individuals. Furthermore, only activity showed an intercept–slope interaction: Crickets with high levels of activity did not increase their activity levels with age as much as less active crickets. This suggests that some crickets are approaching an upper limit to how active a cricket can be. Moreover, it suggests that while crickets change with age in tendency to leave the tube, shyness, and exploration, they do not change greatly relative to each other, and so measures at one point in life are relevant to life-history or fitness measures that are measured over a lifetime.

The amount of variation of intercepts compared to the mean intercept was greater than the among-individual variance in slopes compared to the average slope. Therefore, all traits show a very limited level of among-individual variation in the way they change with age. Coefficients of variation are not valid as the slopes can be negative, but it seems reasonable that a simple comparison between the mean and variance of an effect can be made. We were unable to test this statistically as the deviance information criterion of Poisson models in MCMCglmm is not focused correctly (Hadfield 2012), preventing us from comparing models with and without the random slope term. Dingemanse et al. (2012) considered similar estimates of among-individual variance in slopes to show a lack of individual-specific change. We do not think that the lack of among-individual variation in slopes reflects a lack of power in our study, as the 95% CRIs are narrow (Martin et al. 2011). Therefore, the way the crickets’ traits changed with age is not individual specific, but governed by over-arching population-wide forces. Previous work has indicated that individuals can consistently differ in their plastic response to environmental gradients and suggested that age could be viewed as another element of the environment (Nussey et al. 2007; Dingemanse et al. 2010). Our results suggest that, at least in wild crickets, how behavioral traits change with age are not distinct traits in their own right.

### Increases in among-individual variance with age

The estimates of the change in among-individual variance over time from the RRs for each trait showed identical patterns, with a drop in among-individual variance towards the mean age, followed by a large increase towards later life. This partly supports our prediction that the patterns of variance change will follow that of behavioral traits in humans (Roberts and Del Vecchio 2000) and great tits (Dingemanse et al. 2012). However, the curves also had properties of the U-shaped curves found in the additive genetic variance of nonbehavioral traits in *Drosophila* (Tatar et al. 1996) and mute swans (Charmantier et al. 2006). Overestimates of the variances at the extremes are likely (Promislow et al. 1996), so the underlying pattern is perhaps more likely to be a monotonic increase with among-individual variance over time than U-shaped.

A common assumption is that residual variance is consistent over time, however if it either consistently rises or falls, then estimates of repeatability will not follow the same pattern (Brommer 2013a). Our estimates of residual variance at different captures only show such a pattern for activity, with a decrease with increasing test number. Despite this, estimates for repeatability still follow an approximately U-shaped pattern, consolidating the findings from the RRs. Assuming that among-individual variance is related to additive genetic variance, this suggests that the degree of heritability changes over adult lifetime (Tatar et al. 1996; Charmantier et al. 2006; Brommer 2013a). Therefore, selection could have different outcomes depending on the age of the individual. Relatively few studies have estimated gene by environmental interactions (G × E) in wild animals, and whether this change in among-individual variance is reflected in expression of additive genetic variance with age deserves investigation (Brommer 2013a).

In humans, behaviors become more repeatable as individuals age (Roberts and Del Vecchio 2000). This is thought to result from reinforcement of individual behavior (Roberts and Del Vecchio 2000). Individual experience resulting from being more or less risk-prone or active (e.g., exploring away from the burrow and finding food or potential mates) has the potential to reinforce such behaviors in crickets, perhaps leading to an increase in consistency. For shyness and exploration, no such clear pattern emerged from our models with separate estimates of repeatability at each of the captures. This appears to be driven by the very wide CRIs for among-individual variance at each time point for each trait. Only one of the estimates for shyness and none for exploration were significantly different from zero. Such “character-state” approaches are more “data hungry” than RRs (Brommer 2013a), perhaps limiting our ability to detect equivalent patterns, except if they are strong (e.g., for activity).

### Population-level effects

Crickets left the tube more often and faster, moved about, and explored their environment more as they got older. An increase in activity has been reported in the Siberian dwarf hamster, *Phodopus sungorus* (Kanda et al. 2012), but this contrasts with age-related declines in activity that have been reported in other insects such as the fruit fly, *Drosophila melanogaster* (Le Bourg and Lints 1992), and the housefly, *Musca domestica* (Sohal and Buchan 1981). Increasing activity as they age would allow crickets to range beyond their immediate environment to contact new mates, once they have mated (or chosen not to) with their neighbors. This suggests that in wild field crickets reproductive value is maximized in older age classes by greater risk taking, possibly because the greater residual reproductive value of young crickets favors the lower risks associated with being less active (Williams 1966; Hirshfield and Tinkle 1975; Wolf et al. 2007).

The effect of selective disappearance (the fixed effect of maximum age) was not important, so crickets that lived longer did not have specific levels of boldness. This appears to support the model of Luttbeg and Sih (2010), who suggest that risk-taking individuals will not suffer a reduced lifespan. This is because the risk-taking individuals will be in a better state, and better able to avoid predators. Summing the effects of selective disappearance and within-individual change with age gives the among-individual change with age (van de Pol and Verhulst 2006). Therefore, by adding the coefficients of the fixed effect of maximum age (which models selective disappearance) and the fixed effect of age (which models the within-individual change with age), we find values with the same sign as the fixed effect of age (0.051, −0.020, 0.027, and 0.019 for tendency to leave the tube, shyness, activity, and exploration, respectively). Therefore, the effect of among-individual differences in age on a trait followed the same direction as the within-individual change with age, and differences within an individual with age (e.g., a decrease in shyness) are mirrored at the population level (older individuals being less shy than younger individuals).

In all models, the fixed effect of test number was not important. Therefore, crickets did not become habituated to the assay. It has been shown elsewhere that crickets show a “forgetting curve” (Yano et al. 2012), which implies that experiences 3–4 days in the past do not affect behavior. Returning the crickets to the wild between captures, and spacing recaptures by around 10 days, was clearly sufficient to prevent habituation.

Furthermore, we did not find any effect of mass. Size has been suggested to not be a good indicator of condition in this species (Rodríguez-Muñoz et al. 2010), and so may have limited bearing on the traits we measured. Finally, males were less active than females and tended to be less likely to leave the tube, were shyer, and less exploratory. In this species, males typically sing at a burrow while females move among them, exercising mate choice (Rodríguez-Muñoz et al. 2010). This difference in the role of the sexes could be driving these differences in personality (Schuett et al. 2010).

### Correlations

We found residual correlations between exploration and shyness and exploration and activity. Residual correlations can result from correlated individual plasticity, correlated measurement error, or driven by unmeasured internal or external effects (Brommer 2013c; Dingemanse and Dochtermann 2013). We controlled for environmental effects by conducting our assays in standardized laboratory conditions and included temperature of the laboratory in all our models. We also used an automated system that did not change over time to measure our behaviors. Therefore, we should avoid Brommer’s “individual gambit” when concluding that any residual correlation suggests a within-individual correlation (Brommer 2013c; Dingemanse and Dochtermann 2013; Araya-Ajoy and Dingemanse 2014). Therefore, the significant residual correlations between exploration and both shyness and activity suggest that the traits are correlated at the within-individual level. This means that a cricket is limited in its ability to be both exploratory and shy when it suits it. A correlation between activity and exploration could “artificially” emerge if crickets that are very active end up moving about in different parts of the test arena in the first minute of the trial, giving them a high exploration score. However, there was no among-individual correlation between these traits, suggesting that more active individuals are not more exploratory, and so the within-individual correlation is genuine. Within-individual correlations indicate that the traits are influenced by a central mechanism that varies among individuals, creating individuals that are both different from each other and constrained to behave in particular ways (Stamps 1991; Sih et al. 2004) according “evolutionary characters” (Wagner 2001) and “behavioral characters” (Araya-Ajoy and Dingemanse 2014). Implicit to the evolutionary/behavioral characters framework is the assumption that this association between shyness and activity is adaptive (Bell and Sih 2007), a prediction that should be tested empirically (Araya-Ajoy and Dingemanse 2014).

## CONCLUSION

Overall, we found evidence for individual-specific behaviors that are consistent over adult lifetimes. We did not however find strong evidence for individual-specific changes in behavior over time. This suggests that there are constraints on how individuals change over time, for example, for all crickets, an increase in activity with age is beneficial or unavoidable. All traits showed a similar pattern in among-individual variance, with an increase later in life, with implications for the effect of selection at different ages. Finally, we found significant correlations between shyness and exploration and between exploration and activity within individuals, suggesting correlated plasticity within individuals in the expression of these traits and so a degree of nonindependence in expression and evolutionary history.

## SUPPLEMENTARY MATERIAL

Supplementary material can be found at http://www.beheco.oxfordjournals.org/

## FUNDING

Funding for this research was provided by National Environment Research Council (studentship number: NE/H02249X/1; grant number: NE/H02364X/1), the Leverhulme Trust, and a Royal Society Fellowship to T.T.

## Supplementary Material

Supplementary Data
