# Supplementary material for: Dynamics of among-individual behavioral variation over adult lifespan in a wild insect
Source: Behav Ecol. 2015 Apr 29;26(4):975–85. doi: 10.1093/beheco/arv048 (PMC4495759; doi:10.1093/beheco/arv048)
Supplement: Supplementary Data [file supp_26_4_975__index.html]

Dynamics of among-individual behavioral variation over adult lifespan in a wild insect — Dynamics of among-individual behavioral variation over adult lifespan in a wild insect — Supplementary Data 

# Dynamics of among-individual behavioral variation over adult lifespan in a wild insect

## Supplementary Data

Data files

**Files in this Data Supplement:**

- Supplementary Data - Supplementary Data
