# Supplementary material for: Dynamics of among-individual behavioral variation over adult lifespan in a wild insect
Source: Behav Ecol. 2015 Apr 29;26(4):975–85. doi: 10.1093/beheco/arv048 (PMC4495759; doi:10.1093/beheco/arv048)
Supplement: Supplementary Data [file supp_arv048_DF_Pers___age_in_wild_crickets_BE_2_supp_mat2.docx]

# Supplementary materials for Dynamics of among-individual behavioral variation over adult lifespan in a wild insect

Model formulae

Random regression, = the traitvalue of individual *i* at time *j*:


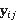

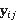

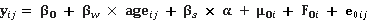


β_0_=mean at age 0, β_w_ x age_ij_=within individual change with age, β_s_ x α =effect of selective disappearance, μ_0i_=individual specific mean, F_0i_= fixed effects & e_0ij_=residual variance at time point *j*. The effect of among-individual differences of age of last test on a trait (β_B_) can be found by summing β_w_ and β_s_.

Variance in crickets at different recaptures, =valueof individual *i*:


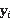

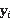

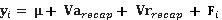


μ is the intercept, Va_recap_&Vr_recap_=among-individual and residual variance respectively at specific recaptures, F*_i_*=fixed effects.

Bivariate models, z & y values for individual *i*:


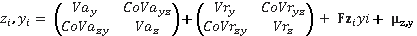


Va=among-individual variance, CoVa=among-individual covariance, Vr=residual variance, CoVr=residual covariance, F_i_ = fixed effects. Separate intercepts and the fixed effects have independent effects on the traits.

Supplementary table

Table S1. Full results for multivariate mixed-model of shyness, activity and exploration.Given are the posterior distribution modes (PDM) and 95% credible intervals (CRIs) of the variance, covariance or correlation estimated. Correlations and fixed effects are considered significant if the 95% CRIs do not cross zero (highlighted in bold). Fixed effect names underlined.

|  |  | *PDM* | *Lower 95% CRI* | *Upper 95% CRI* | *pMCMC* |
| --- | --- | --- | --- | --- | --- |
| *Among-individual variances* | Shyness | 0.199 | 0.060 | 0.345 | NA |
|  | Activity | 0.080 | 0.033 | 0.125 | NA |
|  | Exploration | 0.130 | 0.034 | 0.722 | NA |
| *Among-individual covariances* | Shyness & Activity | -0.024 | \| -0.076 \|  \| \| --- \| --- \| | 0.044 | NA |
|  | Shyness & Exploration | 0.050 | -0.143 | 0.206 | NA |
|  | Activity & Exploration | -0.022 | -0.109 | 0.102 | NA |
| *Among-individual correlations* | Shyness & Activity | -0.153 | -0.669 | 0.327 | NA |
|  | Shyness & Exploration | 0.179 | -0.466 | 0.834 | NA |
|  | Activity & Exploration | 0.064 | -0.772 | 0.575 | NA |
| *Residual variances* | Shyness | 1.182 | 0.990 | 1.364 | NA |
|  | Activity | 0.131 | 0.080 | 0.171 | NA |
|  | Exploration | 4.125 | 3.586 | 4.780 | NA |
| *Residual covariances* | Shyness & Activity | -0.031 | -0.090 | 0.045 | NA |
|  | Shyness & Exploration | -0.586 | -0.839 | -0.360 | NA |
|  | Activity & Exploration | 0.334 | 0.217 | 0.469 | NA |
| *Residual correlations* | Shyness & Activity | -0.054 | -0.238 | 0.113 | NA |
|  | **Shyness & Exploration** | **-0.288** | **-0.370** | **-0.167** | **NA** |
|  | **Activity & Exploration** | **0.501** | **0.337** | **0.629** | **NA** |
| *Fixed effects - Shyness* | Age | -0.017 | -0.032 | 0.001 | 0.067 |
|  | Mass | -0.194 | -0.936 | 0.546 | 0.560 |
|  | **Temperature** | **-0.159** | **-0.269** | **-0.039** | **0.010** |
|  | Sex | -0.181 | -0.410 | 0.003 | 0.059 |
|  | Test | -0.014 | -0.135 | 0.082 | 0.707 |
|  | Maximum age | 0.003 | -0.006 | 0.012 | 0.482 |
|  | Year | -0.214 | -0.428 | 0.070 | 0.138 |
| *Fixed-effects - Activity* | **Age** | **0.010** | **0.003** | **0.019** | **0.003** |
|  | Mass | -0.093 | -0.413 | 0.364 | 0.938 |
|  | **Temperature** | **0.141** | **0.089** | **0.199** | **< 0.001** |
|  | **Sex** | **-0.121** | **-0.227** | **-0.020** | **0.019** |
|  | Test | 0.019 | -0.031 | 0.073 | 0.461 |
|  | Maximum age | 0.001 | -0.003 | 0.006 | 0.661 |
|  | **Year** | **-0.586** | **-0.699** | **-0.458** | **< 0.001** |
| *Fixed effects -  Exploration* | Age | 0.007 | -0.021 | 0.038 | 0.607 |
|  | Mass | 0.291 | -1.010 | 1.594 | 0.702 |
|  | **Temperature** | **0.450** | **0.232** | **0.649** | **< 0.001** |
|  | Sex | -0.068 | -0.484 | 0.246 | 0.516 |
|  | Test | -0.015 | -0.229 | 0.162 | 0.765 |
|  | Maximum age | -0.002 | -0.015 | 0.017 | 0.906 |
|  | **Year** | **0.540** | **0.073** | **0.973** | **0.022** |
